# Biological Correlates of Post-Traumatic Growth (PTG): A Literature Review

**DOI:** 10.3390/brainsci13020305

**Published:** 2023-02-10

**Authors:** Liliana Dell’Osso, Barbara Carpita, Benedetta Nardi, Chiara Bonelli, Martina Calvaruso, Ivan Mirko Cremone

**Affiliations:** Department of Clinical and Experimental Medicine, University of Pisa, 56126 Pisa, Italy

**Keywords:** post-traumatic growth, post-traumatic stress disorder, trauma, biological correlates, neurobiology

## Abstract

Since the beginning of medical science, much research have focused on the psychopathological effects of traumatic experiences. Despite in past centuries the scientific literature on mental health has been mainly focused on the harmful effects of traumatic occurrences, more recently the idea of “post-traumatic growth” emerged, on the basis of a growing interest in the characteristics of resilience and possible positive consequences of trauma. In this framework, increasing attention is now being paid to the psychological meaning of PTG, with a consistent number of psychopathological and epidemiological studies on this subject, but limited literature focused on neurobiological correlates or eventual biomarkers of this condition. The present work aimed to summarize and review the available evidence on neurobiological correlates of PTG and their psychological and clinical meaning. Results highlighted a variety of biochemical and neurobiological differences between PTG and non-PTG individuals, partially corroborating findings from earlier research on post-traumatic stress disorder (PTSD). However, although promising, findings in this field are still too limited and additional studies on the neurobiological correlates of traumatic experiences are needed in order to gain a better understanding of the subject.

## 1. Introduction

In line with the historical development of psychiatry, medical research, up to these days, was mainly focused on the ability of traumatic events to exacerbate a psychopathological picture [1], without paying attention to the possible positive effects deriving from it. While there is an overwhelming amount of literature on the negative physical and psychological consequence of trauma, the studies focusing on the positive aspects are still scant. In recent times, growing attention has been given to the concept of “resilience” and subsequently to the associated issue of the possible positive consequences of trauma, acknowledging the already present literature on the matter [2,3,4,5,6] and leading to the conceptualization of the so-called post-traumatic growth (PTG) [7].

PTG was first described by Tedeschi and Calhoun [8,9] as positive psychological changes that can arise in relation to responses to a wide set of traumatic experiences—such as surviving a natural disaster [10], a terroristic attack [11], or cancer [12]—and the highly emotional struggle that comes with them. Interestingly, the authors also argued that the traumatic events by itself would not be sufficient to cause PTG, but for the latter to manifest, individuals must reflect on the experience and seek to find meaning in them. From this viewpoint, the growth would come from the integration of the trauma and from a brand-new sense of the world [13]. PTG has been reported to occur in five major areas such as increased appreciation for life, more meaningful relationships, increased sense of personal strength, identifying new priorities, and a richer existential and spiritual life [14], with no differences based on culture and context [15,16]. In this new framework, Tedeschi and Calhoun developed the only validated scale for measuring PTG: the Post-Traumatic Growth Inventory (PTGI) [14]. The PTGI consist in 21 items divided in five domains: (I) *Relating to Others*, (II) *New Possibilities*; (III) *Personal Strength*, (IV) *Spiritual Change* and (V) *Appreciation of Life*. Each item is assigned a score from 0 (*I did not experience this change as a result of my crisis*) to 5 (*I experienced this change to a very great degree as a result of my crisis*). The questionnaire showed a great internal consistency (*α* = 0.90) and an acceptable test-retest reliability *r* = 0.71.

While growing attention is now being posed on the psychological meaning of PTG, with an increasing number of psychopathological and epidemiological studies on this subject, not many data are available on its biochemical and neural correlates as well as its biomarkers. To date, many studies investigated the correlates of post-traumatic stress (PTS) and post-traumatic stress disorder (PTSD) [17,18], highlighting the involvement of specific brain structures such as the hippocampus [19,20] and the prefrontal cortex [21,22], as well as changes in catecholamines [23] and cortisol [24] levels. Even though similar or opposite modifications on brain structure or activity and biochemical changes are conceivable also in PTG, only few studies have focused on the topic. Thus, the purpose of our paper is to provide an overview of the studies currently present concerning the possible biochemical and neuronal correlates present in PTG and their psychological and clinical meaning.

## 2. Available Literature on Biological Correlates of PTG

One of the first studies acknowledging the clinical consequences on cognitive functioning in PTG was led in 1999 by Eren-Koçak [25]. The authors assessed 53 survivors of the Marmara Earthquakes of 1999, enrolled 3 and 4 years after the earthquakes, and scientifically validated PTG as an independent construct associated with cognitive functioning, in particular the executive functions, and with a peculiar correlation with the *personal growth* domain. The survivors were assessed with the Traumatic Stress Symptom Checklist (TSSC) for traumatic symptoms, the Beck Depression Inventory (BDI) for depressive symptoms, and with the PTGI. Cognitive functions were grouped into three different domains (named memory, executive functions, and processing speed) measured with the Auditory Verbal Learning Test (AVLT), the Rey-Osterreith Complex Figure Test (ROCFT), the Color Trail Making Test, the Short Category Test, the Stroop test and the Verbal Fluency Test. Results showed that *personal growth* domain was significatively positively correlated with higher performance in visual recall, better verbal fluency performance in human names, and less errors in short category test; while, the total score on the PTGI and on the relational growth items had no significant correlations with none of the three domains of the cognitive functions [25].

The first study that supported and revealed, albeit indirectly, the existence of a correlation between PTG and specific brain structures was led in 2002 by Ochsner et al. [26]. A functional magnetic resonance imaging (fRMI) was used to evaluate brain activity alteration in 15 healthy right-handed female subjects who were asked to increase, maintain, or decrease their emotional response to negative pictures. Results showed how cognitive reappraisal of highly negative photographs resulted in a reduction in the subjective experience of negative affect, in an increasing activation of the brain activity in the lateral and medial prefrontal cortex (PFC) and in a decreasing activation in the medial orbito-frontal cortex and in the amygdala. These results may support the hypothesis that PFC was involved in cognitive reappraisal, a process by which feelings and thoughts may be modulated to reach a PTG [26].

Some years later, Rabe et al. [27] investigated the correlation between the PTG and the brain function in a sample of 82 survivors of severe motor vehicle accidents (MVAs), who completed the German version of the Posttraumatic Growth Inventory (PTGI) and the German version of the Positive and Negative Affect Schedule (PANAS). PTS symptoms were assessed by the German version of the Clinician-Administered PTSD Scale (CAPS) [27]. Brain activity was measured using resting electroencephalogram (EEG); the results showed that the total score on the PTGI were significantly and positively correlated with the frontocentral EEG alpha asymmetry, suggesting an association between PTG and higher relative left cortical activity. Noticeably, the spiritual changes domain was the only one not correlated with the relative left frontocentral activity. Results also highlighted that the association between PTG and left frontocentral activity was not significantly altered when controlling for Trait Positive Affect (T-PA) score. As stated by the authors, these findings seem to suggest that the positive correlation between PTG and relative left frontocentral activity was independent from T-PA, supporting the hypothesis that PTG did not represent only a dispositional positive effect but reflected an active approach toward achieving new goals and perspectives for the self, which was suggested to be underlain by a higher relative left frontal brain activity [27] (See Table 1).

More recently, another study focused on the relationship between PTG and basal whole-brain functional connectivity, using resting-state functional magnetic resonance imaging (resting-state fMRI) [28]. A sample of 33 subjects were asked to choose a stressful or traumatic life event experienced in a list of ten different “low magnitude” events, then the Japanese versions of PTGI, IES-R, and BDI were used to quantify respectively PTG, post-traumatic symptoms, and depression symptoms. After selecting eight areas corresponding to different functional networks of the brain, results showed that PTGI scores were significantly and positively correlated with brain activation in the rostral PFC (rPFC) and in the superior parietal lobule within the left central executive network (CEN). The rPFC activation within the CEN suggested that patients with higher PTG had more positive functional alterations in the prospective memory, while the superior parietal lobule (SPL) activation suggested a positive correlation between PTG and beneficial effects in the working memory. In addition, the seed-based analysis reported that the PTGI scores were significantly and positively correlated with a stronger connectivity between the SPL seed and the supramarginal gyrus (SMG), one of the brain regions associated with the ability to reflect on mental states of others. The authors stated that their results supported the presence of a stronger connectivity between memory functions and social functioning among subjects with PTG, leading to hypothesize that their better sociality may be linked to an increased use of memory for reflecting on other mental states during social interactions [28].

Anders et al. [29] used magnetoencephalography (MEG) on veterans who had been exposed to traumas of different severity, reporting or not a PTSD diagnosis (N = 106 vs. N = 193, respectively). Post-traumatic symptoms were assessed using the Clinician Administered PTSD Scale (CAPS) or the Structured Clinical Interview (SCID); the Deployment Risk and Resilience Inventory (DRRI) was used as an indicator of the lifetime exposure to trauma, and PTG was investigated with the PTGI. MEG detected the synchronous neural interactions (SNIs), consisting in two levels defined global (GSNI) and local (LSNI) and identifying as markers of the brain activity. There was a significant difference in the total score on DRRI between the control and the PTSD groups, but not in the total score on the PTGI. Moreover, the PTG/trauma ratio differed significantly between the two groups: control veterans presented more growth per trauma than PTSD veterans. In the control group the total score on the PTGI was significantly and negatively associated with the global synchronous neural interactions (GSNIs) in both hemispheres; no significant correlation was found instead in the PTSD group. Furthermore, among controls, the highest decreases of SNIs with the increase of PTGI score were reported between the left and right parts of the medial PFC (mPFC), while the reduction of parieto-occipital cortex SNIs with PTGI score increase was more evident in the left than in the right hemisphere. These downward modulations of the neural network could be hypothesized to underlain PTG, and in particular the positive cognitive processing and restructuring that occur after trauma. In this framework, the authors highlighted that, considering the key role of mPFC for the modulation of fear behavior, downward modulations of SNIs in this area may reduce fear expression or conditioned response to fear, eventually facilitating PTG [29]. 

Nagawa et al. [30] focused instead on the correlation between PTG and the variation of regional gray matter volume (rGMV) in the dorsolateral PFC (DLPFC) using voxel-based morphometry [30] in a sample of 26 students who experienced the East Japan Great Earthquake occurred on March 2011. In particular, the authors measured the difference between the rGMV measured before and 3 months after the event. PTG was investigated with the Japanese version of the PTGI, depressive symptoms were assessed using the Japanese version of the Center for Epidemiologic Studies Depression Scale (CES-D), anxiety was evaluated with the Trait Anxiety (T-A) subscale of the Japanese version of the State-Trait Anxiety Inventory (STAI), general intelligence was measured using Raven’s Advanced Progressive Matrox (RAPM), post-traumatic symptoms were assessed using the CAPS. Finally, the Mini-International Neuropsychiatric Interview (M.I.N.I) was employed for excluding neuropsychiatric disorders. Results revealed that, after controlling for the score reported at the other scales and for socio-demographic variables, the PTG total and *relating to others* domain scores were positively associated with an increased regional grey matter volume (rGMV) in the right DLPFC after the disaster. These results support the authors’ hypothesis that alteration on DLPFC, a region already known for its role in resilience and coping mechanisms in PTSD studies [32,33], would be a key neural correlate of PTG. Moreover, the students with lower PTGI *relating to others* scores reported a reduced rGMV in the DLPFC. The authors hypothesized that this finding could be a neurobiological correlate of a reduced sense of connection with other people. Meanwhile, according to the multiple regression analysis performed by the authors, CAPS total score was also significantly, but negatively, correlated with the peak of the statistically significant delta-rGMV in the right DLPFC, eventually supporting the hypothesis that PTG and post-traumatic symptoms could co-occur in the same subjects [30,31,32]. 

A further research Investigated the changes of heart rate variability (HRV) and the activation of the DLPFC, involved in the emotion regulation [34], in response to the presentation of negative, neutral, and positive pictures correlated with PTG [31]. The sample was composed of 90 people who were in the central and surrounding areas of the container explosions at the Port on Tianjin in August 2015, divided into three groups: a control group, a PTSD group, and a PTG group, according to the scores obtained in the PTSD Checklist-Civilian Version (PCL-C) questionnaire and in the PTGI. Results showed that while showing positive images, the low frequency and high frequency component of HRV was significantly higher in PTG group than in control and PTSD group, but no difference was showed between PTSD group and control group. No differences were reported among the three groups for the presentation of neutral or negative stimuli. According to the authors, these results may suggest an increased efficiency in processing emotional stimuli in the PTG group. Concerning the DLPFC activation, results highlighted that the oxygenated hemoglobin level of left DLPFC was higher in PTG group than in the control group in response to negative pictures, while there was no difference with respect to the PTSD group; on the other hand, while showing positive pictures, oxygenated hemoglobin level of right DLPFC was higher in PTSD group than in the control group, while there was no difference with the PTG group. These results revealed different roles of the right and the left part of DLPFC in the emotion regulation during a posttraumatic experience [31] (See Table 1).

Another branch of research focused on HPA axis and immune system alterations after a traumatic event and a traumatic growth. A preliminary study in the field was based on the hypothesis that Cognitive-Behavioral Strategies (CBS) therapy, aiming to ameliorate trauma processing and PTG, could lower cortisol levels [35]. The results showed that participants with PTSD who underwent CBS therapy demonstrated lower levels of cortisol and higher levels of cognitive-behavioral stress management, in terms of PTG, which the authors measured through Benefit Finding scale (BFS) to assess positive contributions, and through an abbreviate version of the Profile of Mood State Scale (POMS) to assess mood on several dimensions during the past week [35]. Some years later, Smyth et al. [36] specifically analyzed cortisol serum levels in a sample of PTSD patients assessed with the PTGI. Results highlighted how subjects with higher PTGI scores, especially in the *appreciation of life* and *new possibilities* domains, also had lower salivary cortisol levels, suggesting an inverse correlation between the two [36]. A similar research evaluated the correlation between cortisol and PTG in 99 patients with breast cancer, by measuring cortisol slope at waking up in morning, noon, and at 5 and 9 p.m. [37], and reported how PTGI scores were inversely proportional to diurnal cortisol (See Table 2). 

On the same line, several studies have identified a relationship between immune system states and the processing of stressful events [38,39,40,41,42,43]. Particularly, a positive reaction to a stressful event was reported to eventually exert a beneficial effect on the immune response. In 1994, Stone et al., observed that a positive response to stress led to an increased mucosal secretion of IgA, thus potentiating the immune system [40]. Bower and Fahey [39], while analyzing a group of HIV-positive men who had recently experienced the loss of a friend due to HIV, reported that a cognitive processing on the loss meaning guaranteed the maintenance of average CD4 levels for 2–3 years, while those who failed the cognitive processing lost an average of about 119–155 CD4 [39]. More recently, two major works underlined a strong link between PTG and the immune system. The first evaluated a sample of 41 patients with hepatocellular carcinoma (HCC), measuring peripheral blood leukocytes and survival at t0 and follow-ups. Results highlighted that subjects with higher scores of PTGI (and above all higher scores in the *spirituality*, and *marital support* domains) showed higher levels of lymphocytes and survived longer than participants with PTG scores below the median [44]. At baseline, a higher lymphocyte count was reported only among females with PTG *spirituality* domain score above the median, while at follow-ups associations between PTG spirituality domains and leukocytes were found in both genders, being detectable also for *personal strength* and *relating to others* domains (3- and 6-month follow-up, respectively) [44]. Another research led on 412 HIV patients reported more controversial results. The authors did not find an association between PTG and viral load in the whole sample, although an inverse correlation between viral load and PTG was reported among subjects with lower pessimism levels as measured by the revised Life Orientation Test. Moreover, PTG was positively associated with CD4 counts only among Hispanic subjects and among subjects with lower optimism scores. Globally, these results may suggest an impact of cultural and personal factors on the beneficial effects of PTG [45] (See Table 3). 

Considering the role of oxytocin in trauma, a recent review underlined the importance of this hormone in determining resilience, and thus eventually PTG. Oxytocin was reported to be involved in attenuating the neurophysiological and neurochemical effects of trauma in the brain, determining a better psychological/social attachment, and thus increasing resilience to subsequent traumatic events. Moreover, patients diagnosed with PTSD received therapeutic benefit from oxytocin administration, which could be moderated by other factors such as other comorbid mental disorders, adverse childhood experiences, perceived environmental safety, presence of support, attachment style or oxytocin receptors’ polymorphisms [46,47].

Lastly, a relatively new branch of research focused instead on the genetic aspects of PTG. In particular, a study from Dunn [48] was the first to identify gene-environment interaction (GxE) in the context of PTG. The study examined if and how ten common variants in seven genes [BDNF (rs6265, chromosome 11p14), CACNA1C (rs1006737, chromosome 12p13), CRHR1 (rs12944712; chromosome 17q21), FKBP5 (rs1360780, rs9296158, and rs9470080, chromosome 6p21), OXTR (rs53576 and rs2254298, chromosome 3p25), RGS2 (rs4606, chromosome 1q31), SLC6A4 (variable number tandem repeat VNTR and rs25531, chromosome 17.q11–17.q12)] modified the associations between Hurricane Katrina exposure and development of PTG rather than PTSD in a sample of 205 low-income non-Hispanic Black people residing in New Orleans. A specifically tailored scale was employed to measure Hurricane Katrina exposure. Subjects were assessed with the PTGI and the Impact of Events Scale–Revised (IES-R). Results highlighted a significant positive association between PTGI total score and the presence of homozygotes rs4606 variant of RGS2 gene, which was later confirmed after correcting for multiple testing and appeared mostly driven by a GxE interaction, rather than only by a genetic effect. In particular, among participants with low levels of Hurricane exposure, minor allele (G allele) homozygous subjects for rs4606 reported lower total scores on PTGI, while the same variant was associated with higher total scores on PTGI among subjects with moderate and high levels of exposure. Moreover, a significant association between PTG and the variant rs1306780 on the FKBP5 gene was also revealed, with a higher likelihood of experiencing PTG in subjects carrying the T allele. This association seemed to be exclusively driven by the genetic effect, but not survived after multiple testing correction [48]. As stated by the authors, their results further support the link between trauma-related symptoms and RGS2 rs4606 variant, which, according to the literature, is known to be associated with anxiety disorders, behavioral inhibition, and PTSD. Interestingly, another study in Hurricane survivors reported complementary results with respect to the study from Dunn et al. [48], showing greater levels of PTSD among major allele homozygous subjects with high levels of exposure [48,49]. A more recent work by Miller et al. [50] examined the relationship between epigenetic mechanisms, in particular DNA methylation of stress genes nuclear receptor subfamily 3 group C member 1 (NR3C1) and FK06 binding protein 5 (FKBP5), and the different possible biological responses to trauma, including the development of post-traumatic symptoms, PTG or resilience. The authors followed a salutogenic approach, which focuses on health rather than on illness, and considers health as a dimensional continuum [51]. The sample was composed of 47 first-year paramedicine students from two Australian universities, who answered the Posttraumatic Stress Disorder Checklist for DSM-5 (PCL-5), the Brief Resilience Scale (BRS) and the Posttraumatic Growth Inventory X (PTGI-X). Methylation analysis was performed on salivary samples of each student collected via Oragene kits (DNA Genotek); 89 CpG sites within the NR3C1 gene and 52 CpG sites within the FKBP5 gene were investigated for the association with the three different possible responses to trauma (PTG, PTSD, resilience). Results reported a significant positive association between PTSD symptom severity and a total of three CpG sites (2 in FKBP5 and 1 in NR3C1); moreover, the correlation with CpG in the FKBP5 gene (cg07485685) was maintained after multiple testing correction. In particular, a higher severity of PTSD symptoms was significantly associated with the methylation of promotor-associated region cg03906910 of NR3C1, instead lower PTSD symptoms were associated with low to moderate methylation of 2 FKBP5 5′ untranslated regions (UTRs). Lower PTG levels were instead significantly associated with hypermethylation of a non-promotor NR3C1 region and with hypomethylation of a promotor-associated NR3C1 region. Noticeably, NR3C1 and FKBP5 methylation appeared to be associated only with lower PTG levels and not with higher PTG levels. Moreover, lower resilience levels were significantly associated with methylation of three NR3C1 sites, while higher resilience levels were significantly associated with methylation of other two NR3C1 sites. The authors also reported a significant but opposite association between CpG site cg07485685 in gene FKBP5 and both resilience and severity of PTSD symptoms: DNA methylation at this CpG site correlated with greater resilience and with lower severity of PTSD. Globally, a correlation between methylation of different FKBP5 and NR3C1 sites and the three different possible responses to trauma (resilience, PTG and PTSD) was suggested, supporting the hypothesis that epigenome can be modified by traumatic events [50] (See Table 4).

## 3. Discussion

The experience, witness, or fear of significant harm, death, or sexual assault, are univocally considered potentially traumatic events [52]. Even if the majority of the population will be exposed to at least one traumatic experience during lifetime [53,54], only around 6% will develop unfavorable psychological reactions such as PTSD [53]. On the other side, sometimes the experience of a traumatic event, determining or not the onset of post-traumatic symptoms, would lead to the development of resilience or even PTG. Interestingly, is precisely resilience the most common outcome of a traumatic event [55], defined as the ability to “bounce back” and function at pre-trauma levels [56,57] that does not exclude the presence of a co-existing post-traumatic symptomatology? PTG is another reaction to traumatic events, which is gaining more interest in the most recent years. Since its recognition as a distinct and independent disorder, PTSD has always attracted and monopolized a lot of attention in epidemiological, clinical and, more recently, neurobiological studies [58], while potential positive outcomes of traumas such as PTG remain relatively neglected until recent years. However, this research methodology, mainly focused on the pathological side, does not adequately take into consideration a person’s overall mental health or the vast range of potential reactions to traumatic events [59]. More recently, a new salutogenic approach to trauma emerged [51], which recognizes both negative and positive trauma reactions and offers a theoretically valid way to look at the complicated relationship between trauma and various psychological outcomes [59,60]. This approach seems to be in line with the increased tendency toward applying a dimensional approach to psychiatric conditions, stressing the presence of a continuum between hyper-adaptive traits, sub-threshold, and full-threshold manifestations from a psychopathological, neurobiological, and neurodevelopmental viewpoint [61,62,63,64,65,66,67]. In this framework, the purpose of our article was to collect the evidence reported so far of neurobiological modifications associated with PTG. According to the above reviewed literature, to date only a limited number of studies have explored the matter but, interestingly, often reported similar yet opposite modification in PTG compared to those in PTSD, leading to hypothesize that similar factors would be involved in both the conditions. One of the most common alterations reported in PTSD patients was a reduction in the volume of specific brain region [58]; in particular, PTSD patients are known to show a lower volume of the hippocampus and of the ventromedial PFC (VMPFC). Furthermore, recent evidence are reporting the possible reversibility of these abnormalities after treatment with paroxetine [68], the severity of post-traumatic symptoms as an important factor in determining the sizes of the volume differences [69,70], and the eventual role of hippocampal volume as a pre-trauma risk factor for PTSD [69]. Interestingly, when investigating the gray matter volume on PTG patients, similar yet opposite changes where highlighted, in particular reporting an increase in the right DLPFC [30]. Functional neuroimaging studies have revealed altered activity in the amygdala, VMPFC, and dACC, as well as in the hippocampus and insular cortex, in people with PTSD [71,72,73]. Similarly, in PTG subjects, it has recently been reported decreased activation in the medial orbito-frontal cortex and in the amygdala [26] alongside an increase in the PFC and left frontocentral cortical activity [27] as well as in the lateral and medial PFC [26] and the rPFC [27], suggesting positive functional alterations in the prospective memory. Likewise, other studies highlighted different modification on GSNIs in PTSD [74] and PTG [29] subjects. Another interesting parallel study concerns salivary cortisol levels: while several studies investigated the role of PTSD in causing a deregulation of the HPA axis [75,76,77,78], few others acknowledged salivary cortisol level alterations in PTG reporting reduced levels of the same [35,36,37]. Interestingly, even though in the occurrence of PTSD typically much more importance was given to exposure to trauma and its environmental factors at the expense of neurobiological predisposing factors, increased attention is being focused on the genetic and epigenetic contribution [79,80,81,82] thus opening the way for similar investigations on the development of PTG [48]. The limited literature in this field seems promising. The glucocorticoid receptor (NR3C1) gene, which has been connected to pathological consequences of trauma, seemed to be linked to methylation alterations in PTG, consistent with the well-established idea that glucocorticoids are stress hormones [50]. Moreover, minor and major allele of the same RGS2 gene rs4606 variant were intriguingly reported to be respectively associated with PTSD and PTG after trauma exposure in two different studies [48,49]. Finally, it should be noted that a study protocol is currently in progress, aiming to observe the effects of the nature-based walk on PTG and the associated psychophysiological alterations in 246 participants, assessing subjects with the TSS, TSC-40, and PTGI and measuring at baseline and three months after the interventions HRV, Cortisol, C-Reactive protein (CRP), Interleukin-6 (IL-6), and Brain-derived neurotrophic factor (BDNF) levels [83]. 

As previously discussed, a variety of biochemical and neuronal alterations were reported to differentiate PTG from non-PTG subjects in numerous studies, partially confirming results from previous research on PTSD; however, to date, evidence, although promising, are still too scant for allowing us to reach a conclusion on the subject. Genetic and neuroimaging-based investigations seem promising: further studies in the field should be carried out including in the same protocol the investigation of both PTSD and PTG-related alterations, in order to comprehensively assess the subject. In addition, in the framework of an integrative approach between central and peripheral pathways, immunological, and eventually biochemical and metabolic studies appear to be of particular interest in the field of trauma related vulnerabilities and changes [64]. PTSD has been reported to be associated with several other somatic disorders linked to immune alterations, from inflammatory bowed disease to multiple sclerosis, being one of the most recognized models of a psychopathological factor influencing somatic disorders [84]. Investigating the association between PTG and other systems’ alterations or pathologies may shed more light on the complex interactions between central nervous system and the periphery, improving our knowledge on the pathophysiology of both mental and somatic disorders, considered as different manifestations of a same condition. 

Results from this review stress the need of further research on neurobiological correlates of post-traumatic experiences, in order to gain a better understanding of the matter: future studies should be conceptualized in light of a spectrum approach, which would consider pathological and beneficial consequences as two sides of a broader phenomenon. 

## Figures and Tables

**Table 1:** Neuroimaging and cognitive studies.

Reference	Sample	Psychological Assessment	Biological Parameters	Main Findings
Ochsner, 2002 [26]	15 right-handed female subjects; age range = 18–30 years	Subjects were asked to increase, maintain, or decrease their emotional response to negative pictures	The brain activity was evaluated with fRMI	The cognitive reappraisal of highly negative photographs resulted in a reduction in the subjective experience of negative affect, in an increasing activation of the brain activity in the lateral and medial PFC and in a decreasing activation in the medial orbito-frontal cortex and in the amygdala.
Rabe, 2006 [27]	82 survivors (F = 55; M = 30) of MVAs; mean age = 41.54 ±13.19;	PTGI; PANAS; CAPS	The brain activity was measured using resting EEG	The total score on the PTGI resulted significantly and positively correlated with the frontocentral EEG alpha asymmetry and this correlation was not significantly altered when controlling for T-PA score. The spiritual changes domain was the only one not correlated with the relative left frontocentral activity.
Eren-Koçak, 2014 [25]	53 survivors (F = 37; M = 16; mean age = 36.7 ± 13.4) of ’99 Marmara Earthquakes, enrolled 3 and 4 years after	TSSC; BDI; PTGI	AVLT, ROCFT, the Color Trail Making Test, the Short Category Test, the Stroop test and the Verbal Fluency Test	The personal growth domain was significatively positively correlated with higher performance in visual recall, better verbal fluency performance in human names, and less errors in short category test.
Fujisawa, 2015 [28]	33 right-handed, healthy volunteers (F = 21, M = 12); mean age = 21.9 ± 5.7 years;	Subjects were asked to choose a stressful or traumatic life event experienced in a list of 10 different “low magnitude” events; PTGI; IES-R; BDI	The basal whole-brain functional connectivity was measured using resting-state fMRI and eight areas, corresponding to different functional networks of the brain, were selected.	The PTGI scores were significantly and positively correlated with brain activation in the rostral PFC (corresponding with a more positive functional alterations in the prospective memory) and in the superior parietal lobule within the left CEN (corresponding with a beneficial effects in the working memory). The PTGI scores were significantly and positively correlated with a stronger connectivity between the SPL seed and the SMG.
Ander, 2015 [29]	299 U.S. veterans including 193 controls (M = 182 (mean age = 59.69 ± 13.36 years; F = 11, mean age = 41.38 ± 15.42 years) and 106 veterans diagnosed with PTSD M = 96, mean age = 52.84 ± 15.09 years; F = 10 mean age = 42.48 ± 11.51 years)	CAPS; SCID; DRRI; PTGI	The SNIs, consisting in two levels defined GSNI and LSNI, were identified as markers of the brain activity and were detected through MEG.	There was a significant difference in the total score on DRRI between the control and the PTSD veterans. In the control veterans: -there was more growth per trauma than PTSD veterans; -the total score on the PTGI was significantly and negatively associated with the GSNIs in both hemispheres; -the highest decreases of SNIs with the increase of PTGI score were reported between the left and right parts of the mPFC, while the reduction of parieto-occipital cortex SNIs with PTGI score increase was more evident in the left than in the right hemisphere.
Nakagawa, 2016 [30]	26 students who experienced the 2011 East Japan Great Earthquake (M = 21, F = 5)	PTGI; CES-D; STAI T-A subscale; RAPM; CAPS; M.I.N.I.	The voxel-based morphometry was used to evaluate the variation of rGMV in the DLPFC.	The PTG total and *relating to others* domain scores were positively associated with an increased rGMV in the right DLPFC after the disaster. The students with lower PTGI *relating to others* scores reported a reduced rGMV in the DLPFC. The CAPS total score was significantly, but negatively, correlated with the peak of the statistically significant delta-rGMV in the right DLPFC.
Wei et al. 2017 [31]	90 people who were in the central and surrounding areas of the container 2015 explosions at the Port on Tianjin (M = 53, F = 37; mean age = 28.58 ± 6.52 years)	Presentation of negative, neutral and positive pictures correlated with PTG; PCL-C; PTGI	HRV activation of the DLPFC	Concerning HRV: -showing positive images, the low frequency and high frequency component of HRV was significantly higher in PTG group than in control and PTSD group, but no difference was showed between PTSD group and control group; -no differences were reported among the three groups for the presentation of neutral or negative stimuli. Concerning the DLPFC activation: -oxygenated hemoglobin level of left DLPFC was higher in PTG group than in the control group in response to negative pictures, while there was no difference with respect to the PTSD group; -in response to positive pictures, oxygenated hemoglobin level of right DLPFC was higher in PTSD group than in the control group, while there was no difference with the PTG group.

PTGI: Post-Traumatic Growth Inventory; PANAS: Positive and Negative Affect Scale; CAPS: Clinician Administered PTSD Scale; TSSC: Traumatic Stress Symptom Checklist; BDI: Beck Depression Inventory; Auditory Verbal Learning Test; ROCFT: Rey-Osterreith Complex Figure Test; IES-R: Impact of Event Scale—Revised; DRRI: Deployment Risk and Resilience Inventory; STAI: State-Trait Anxiety Inventory; RAPM: Raven’s Advanced Progressive Matrox; PCL-C: PTSD Checklist-Civilian Version.

**Table 2:** Studies on HPA axis alteration.

Reference	Sample	Psychological Assessment	Materials and Procedures	Main Findings
Epel, 1998 [38]	A cohort of women exposed to stress	PTGI -	total cortisol response each day and adaptation across days	Women who quickly adapted to the repeated laboratory stressors were more likely to report thriving in response to previous traumatic stress
Cruess, 2000 [35]	34 stage 1 or 2 breast cancer patients enrolled 8 weeks after surgery	CBS therapy; BFS; POMFS	Cortisol levels measured in serum through radioimmunoassay	Participants with PTSD who underwent CBS therapy demonstrated lower levels of cortisol and higher levels of cognitive-behavioral stress management
Smyth, 2008 [36]	25 PTSD patients	PSS-I; PTGI	Salivary cortisol level measured at the arrival, before imagery-based trauma re-experience, after the re-living of the experience	Subjects with higher PTGI scores, also had lower salivary cortisol levels, suggesting an inverse correlation between the two
Diaz, 2014 [37]	99 patients with breast cancer	PTGI	Cortisol slope at waking up in morning, noon and at 5 and 9 p.m for two consecutive day	PTGI scores were inversely proportional to diurnal cortisol.

CBS: Cognitive Behavioral Strategies; BFS; Benefit Findings Scale; POMFS; Profile of mood State Scales; PSS-I: PTSD symptoms interview; PTGI: Post-traumatic Growth inventory.

**Table 3:** Immunological studies.

Reference	Sample	Psychological Assessment	Materials and Methods	Main Findings
Bower, 1998 [39]	40 HIV-positive men, 215 men who recently lost of a friend due to HIV	TGI; CESD; TMAS; MACS interviewS; NHAPS	absolute number of CD4 T lymphocytes per cubic millimeter of peripheral blood	Cognitive processing on the loss meaning guaranteed the maintenance of average CD4 levels for 2–3 years A failure in cognitive processing resulted in a loss of about 119–155 CD4
Stone, 1994 [40]	96 couples	PANAS, MAC	concentration of slgA via radial immunodiffusion, slgA antibody activity to the rabbit albumin via ELISA	Positive affect related directly to slgA, and negative mood related inversely to same-day slgA
Kawamura, 2001 [41]	1550 Japanese male workers	IESR; ECL	Natural killer cell activity, lymphocyte subset counts, and production of interferon gamma and interleukin-4 via phytohemagglutinin stimulation	Count of immunity cells was significantly lower in 12 male with PTSD
Altemus, 2006 [43]	121 subjects of which 16 adult female with PTSD caused by childhood physical and/or sexual abuse		DTH, skin barrier function recovery, circulating numbers of lymphocyte subtypes	Enhanced cell-mediated immune function is in PTSD subjects leading to chronic physiologic and mental stress
Dunigan, 2007 [44]	41 patients with Hepatocellular carcinoma	PTGI	peripheral blood leukocytes and survival at t0 and follow-ups	Higher scores of PTGI correlated to higher levels of lymphocytes
Milam, 2006 [45]	412 HIV patients	LOT	HIV RNA level, CD4 (T helper) lymphocyte count	Viral load and PTG was reported among subjects with lower pessimism levels. Positive association between PTG was and CD4 counts only among Hispanic subjects and among subjects with lower optimism scores

CESD: Center for Epidemiological Studies Depression scale; TMAS: Taylor Manifest Anxiety Scale; PANAS: Positive and Negative Affect Scale; MAC: The Positive and Negative Affect Scale; IESR: Impact of Event Scale-Revised; ECL: Events Check List; DTH: delayed-type hyper-sensitivity; LOT: Life Orientation Test.

**Table 4:** Genetic evidence.

Reference	Sample	Psychological Assessment	Biological Parameters	Main Findings
Dunn, 2014 [48]	205 low-income non-Hispanic Black people exposed to Hurricane Katrina; (F = 197, M = 8; mean age = 25.82 ± 4.39 years)	Specifically tailored scale to measure Hurricane Katrina exposure; PTGI; IESR	There was evaluated the modification in 7 genes: BDNF (rs6265, chromosome 11p14), CACNA1C (rs1006737, chromosome 12p13), CRHR1 (rs12944712; chromosome 17q21), FKBP5 (rs1360780, rs9296158, and rs9470080, chromosome 6p21), OXTR (rs53576 and rs2254298, chromosome 3p25), RGS2 (rs4606, chromosome 1q31), SLC6A4 (variable number tandem repeat VNTR and rs25531, chromosome 17.q11–17.q12).	Results showed a significant positive association between PTGI total score and the presence of homozygotes rs4606 variant of RGS2 gene, which was later confirmed after correcting for multiple testing and appeared mostly driven by a GxE interaction, rather than only by a genetic effect. Among participants with low levels of Hurricane exposure, minor allele (G allele) homozygous subjects for rs4606 reported lower total scores on PTGI, while the same variant was associated with higher total scores on PTGI among subjects with moderate and high levels of exposure. There was revealed a significant association between PTG and the variant rs1306780 on the FKBP5 gene, with a higher likelihood of experiencing PTG in subjects carrying the T allele. This association seemed to be exclusively driven by the genetic effect, but not survived after multiple testing correction.
Miller, [50]	47 first-year paramedicine students (F = 28, M = 18, intersex 1; mean age = 23.43 years)	PCL-5; BRS; PTGI-X	Methylation analysis of stress genes NR3C1 and FKBP5 was performed on salivary sample of each student collected via Oragene kits (DNA Genotek)	Results reported a significant positive association between PTSD symptom severity and a total of 3 CpG sites (2 in FKBP5 and 1 in NR3C1); moreover, the correlation with CpG in the FKBP5 gene (cg07485685) was maintained after multiple testing correction. A higher severity of PTSD symptoms was significantly associated with the methylation of promotor-associated region cg03906910 of NR3C1. Lower PTSD symptoms were associated with low to moderate methylation of 2 FKBP5 5′ untranslated regions (UTRs). Lower PTG levels were significantly associated with hypermethylation of a non-promotor NR3C1 region and with hypomethylation of a promotor-associated NR3C1 region. Noticeably, NR3C1 and FKBP5 methylation appeared to be associated only with lower PTG levels and not with higher PTG levels. Lower resilience levels were significantly associated with methylation of three NR3C1 sites, while higher resilience levels were significantly associated with methylation of other two NR3C1 sites. The authors also reported a significant but opposite association between CpG site cg07485685 in gene FKBP5 and both resilience and severity of PTSD symptoms: DNA methylation at this CpG site correlated with greater resilience and with lower severity of PTSD.

PTGI: Post-Traumatic Growth Inventory; IESR: Impact of Event Scale-Revised; PCL-5: Posttraumatic Stress Disorder Checklist for DSM-5; BRS: Brief Resilience Scale; PTGI-X: Posttraumatic Growth Inventory X.

## Data Availability

Not applicable.
